# Combined interventions to reduce HIV incidence in KwaZulu-Natal: a modelling study

**DOI:** 10.1186/s12879-017-2612-5

**Published:** 2017-07-26

**Authors:** Stéphanie Blaizot, Helena Huerga, Benjamin Riche, Tom Ellman, Amir Shroufi, Jean-François Etard, René Ecochard

**Affiliations:** 1 Service de Biostatistique, Hospices Civils de Lyon, F-69003 Lyon, France; 2 Université de Lyon, F-69000 Lyon, France; 3 Université Lyon 1, F-69100 Villeurbanne, France; 4 CNRS UMR 5558, Equipe Biostatistique-Santé, Laboratoire de Biométrie et Biologie Evolutive, F-69100 Villeurbanne, France; 5 Epicentre, F-75011 Paris, France; 6 Médecins Sans Frontières, Cape Town, South Africa; 7 IRD UMI 233, INSERM U1175, Université de Montpellier, Unité TransVIHMI, F-34000 Montpellier, France

**Keywords:** HIV, Mathematical models, Antiretroviral therapy, Male circumcision, Pre-exposure prophylaxis, South Africa

## Abstract

**Background:**

Combined prevention interventions, including early antiretroviral therapy initiation, may substantially reduce HIV incidence in hyperendemic settings. Our aim was to assess the potential short-term impact of combined interventions on HIV spreading in the adult population of Mbongolwane and Eshowe (KwaZulu-Natal, South Africa) using sex- and age-specific scenarios, and age-targeted interventions.

**Methods:**

A mathematical model was used with data on adults (15–59 years) from the Mbongolwane and Eshowe HIV Impact in Population Survey to compare the effects of various interventions on the HIV incidence rate. These interventions included increase in antiretroviral therapy (ART) coverage with extended eligibility criteria, increase in voluntary medical male circumcision (VMMC), and implementation of pre-exposure prophylaxis (PrEP) among women.

**Results:**

With no additional interventions to the ones in place at the time of the survey (ART at CD4 < 350 and VMMC), incidence will decrease by 24% compared to the baseline rate. The implementation of “ART at CD4<500” or “ART for all” would reduce further the incidence rate by additional 8% and 15% respectively by 4 years and 20% and 34% by 10 years. Impacts would be higher with age-targeted scenarios than without.

**Conclusions:**

In Mbongolwane and Eshowe, implementation of the new South African guidelines, recommending ART initiation regardless of CD4 count, would accelerate incidence reduction. In this setting, combining these guidelines, VMMC, and PrEP among young women could be an effective strategy in reducing the incidence to low levels.

**Electronic supplementary material:**

The online version of this article (doi:10.1186/s12879-017-2612-5) contains supplementary material, which is available to authorized users.

## Background

South Africa has one of the highest HIV prevalence in the world, estimated at 18.8% among adults (15–49 years) in 2012 [1]. However, the HIV epidemic in the country is heterogeneous and KwaZulu-Natal (KZN) has the highest HIV prevalence of the nine provinces in South Africa (estimated at 27.9% in 2012) [1].

UNAIDS set “90-90-90” targets (i.e. 90% of the HIV-positive diagnosed, 90% of those diagnosed under treatment, and 90% of those under treatment virally suppressed) to be achieved by 2020 [2]. However, challenges in the HIV cascade of care remain and, particularly, levels in the cascade vary greatly by sex and age [1, 3–6]. Moreover, voluntary medical male circumcision (VMMC) coverage and demand vary with age [1, 7], with the highest levels found among young individuals. Finally, young women are disproportionately affected by HIV [8, 9] and a prevention intervention such as pre-exposure prophylaxis (PrEP) could be an additional tool to reduce HIV incidence in this subpopulation.

In the present study, we assessed the potential impact of scaling-up antiretroviral therapy (ART) with extended ART eligibility as well as combinations of ART with VMMC and PrEP on HIV incidence among adults (15–59 years old) in Mbongolwane and Eshowe, KZN (South Africa), using age-specific coverage targets as well as age-targeted scenarios, by 4 and 10 years.

## Methods

### Study setting and the Mbongolwane and Eshowe HIV Impact in Population Survey (MHIPS)

Mbongolwane is a rural area in KZN and Eshowe the main town of the municipality. According to the 2011 Census [10], 61,179 people aged 15 to 59 were living in 25,106 households in the area covered by the survey. The KZN Department of Health over the last ten years has conducted an HIV programme in the province that includes HIV testing, HIV care, and ART. Since 2011, in the area of Mbongolwane and Eshowe, Médecins Sans Frontières (MSF) supports the Department of Health HIV and tuberculosis programmes implementing large-scale HIV testing activities and decentralisation of ART initiation, including nurse-initiated and managed ART with the aim of decreasing HIV and tuberculosis incidence, morbidity and mortality.

The Mbongolwane and Eshowe HIV Impact in Population Survey (MHIPS) is a cross-sectional population-based survey conducted from July to October 2013 (i.e. after MSF implementation began). The primary objective of the MHIPS was to assess ART coverage among HIV infected individuals in need of ART in Mbongolwane and Eshowe. The secondary objectives included determining HIV prevalence, HIV incidence, and coverage of HIV services.

The design and procedures of the survey has been previously described [11]. Briefly, a cluster sampling and geospatial random selection was used to identify the households visited. Persons aged 15–59 years living in the area were eligible. Face-to-face interviews were carried out followed by rapid HIV testing on site and blood collection for CD4 count, ART levels, and viral load in HIV-positive cases. Socio-demographic information were collected on all the household members using a household questionnaire and socio-demographic, behavioural, and medical information on each participant were collected using an individual questionnaire.

In total 2377 households were included, 6688 individuals were eligible and 5649 were included (62% were women) [11]. Overall HIV prevalence was 25.2%, higher in women than in men (30.9% vs. 15.9%). Based on ART detection in blood, ART coverage among those eligible according to the national guidelines at the time of the survey was 75.0%, higher in women than in men (78.5% vs. 63.9%) and increased with age (52.2% in younger than 25 years to 84.3% in 35–59 years). ART coverage among all HIV-positive individuals was 53.1%.

### Model and assumptions

A previously developed compartmental model [12, 13] was used to describe HIV transmission, the untreated disease progression, and ART use in the adult population of Mbongolwane and Eshowe, based on the MHIPS data. The model splits the population into compartments according to sex, age (45 one-year strata from 15 to 59 years), and HIV status: HIV-negative (or susceptible) individuals (compartment S), untreated HIV-positive individuals with CD4 cell count >350 cells/mm^3^ (compartment I_1_), untreated HIV-positive individuals with CD4 cell count ≤350 cells/mm^3^ (compartment I_2_), and HIV-positive individuals on ART (compartment T). The model parameters include: i) the force of infection; ii) the “immunosuppression rate”, i.e. the rate at which an individual moves from >350 to ≤350 cells/mm^3^ CD4 cell count; iii) the “treatment rate” or the ART initiation rate; and, iv) the mortality rates. The estimates of the model parameters by sex and age group (15–24, 25–34, 35–59 years) and the estimates of the distributions of the population among the compartments by sex, age, and HIV status were based on the MHIPS data using a methodology described elsewhere [12] (see Additional file 1). We assumed that individuals in compartments S, I_1_, and T had the same risk of death whereas individuals in compartment I_2_ had an additional risk of death due to AIDS, and used mortality rates from another region in KwaZulu-Natal [14]. A sensitivity analysis on values of mortality rates in individuals in compartments I_1_ and T was performed using values of mortality rates among individuals in compartment I_2_ (rather than among HIV-negative individuals): the mortality rates in individuals in compartments I_1_ and T were varied with values being one tenth, one fifth, or half those in individuals in compartments I_2_. Table 1 shows the values of model parameters by sex and age group.

**Table 1:** Infection, immunosuppression, treatment and mortality rates (per 100 person-years) used in the model

Transition rates	Women	Men
Infection rate		
15–24 years	3.0 [2.1; 4.2]	0.3 [0.1; 0.9]
25–34 years	6.6 [4.5; 9.5]	1.9 [0.9; 4.2]
35–59 years	1.0 [0.5; 2.0]	2.2 [1.1; 4.5]
Immunosuppression rate		
15–24 years	15.4 [7.8; 30.4]	13.8 [1.8; 105.4]
25–34 years	6.6 [2.9; 15.1]	25.4 [11.1; 58.4]
35–59 years	12.2 [6.0; 25.1]	13.6 [4.2; 43.8]
Treatment rate		
15–24 years	60.9 [41.5; 89.5]	10.0 [1.3; 76.1]
25–34 years	72.6 [55.1; 95.8]	38.6 [23.9; 62.3]
35–59 years	89.9 [68.3; 118.2]	56.2 [36.4; 86.7]
Mortality rates for individuals in compartments S, I_1_, and T		
15–24 years	0.12	0.16
25–34 years	0.34	0.53
35–44 years	0.23	0.61
45–59 years	0.67	2.20
Mortality rates for individuals in compartment I_2_		
15–24 years	2.52	3.90
25–34 years	6.58	9.85
35–44 years	6.76	5.59
45–59 years	8.71	8.75

The infection, immunosuppression, and treatment rates were estimated using the MHIPS data whereas the mortality rates stemmed from reference [14]. Compartment S: HIV-negative individuals; compartment I_1_: untreated HIV-positive individuals with CD4 cell count >350 cells/mm^3^; compartment I_2_: untreated HIV-positive individuals with CD4 cell count ≤350 cells/mm^3^; compartment T: HIV-positive individuals on ART

Baseline proportions of circumcised men among HIV-negative men by age group were calculated on the MHIPS data: 26%, 21%, and 19% of HIV-negative men were circumcised in the age groups 15–24, 25–34, and 35–59 years respectively (overall: 22%). The protective effect of circumcision was assumed to reduce female-male transmission by 60% [15–17]. We assumed that PrEP was not available and not used at the time of the survey by HIV-negative women. In our PrEP scenarios, the protective effect of PrEP was assumed to reduce male-female transmission by 50% based on meta-analysis results [18].

Information on the population viral load was included to the force of infection (see Additional file 1). In particular, the infectiousness of individuals with viral load <1000 copies/mL in each compartment was assumed to be reduced by 96% [19]. Based on the MHIPS data, the proportion of individuals with viral load <1000 copies/mL was 24% and 14% in compartment I_1_, 5% and 6% in compartment I_2_, and 93% and 92% in compartment T, for women and men, respectively. These values were considered constant over time.

### Interventions modelled

Various interventions were modelled with different scenarios:

- **No change in baseline interventions.** Here, all rates (particularly, treatment and circumcision rates) were considered stable over the simulation time, i.e. equal to the values estimated from the survey. Baseline interventions correspond to the interventions at the time of the survey (i.e. ART at CD4 < 350 and VMMC at 22% coverage among adult HIV-uninfected men).
- **ART intervention.** This intervention explored the impact of changing the ART initiation strategy and increasing the coverage. For this intervention, two ART initiation recommendations were compared: i) the WHO 2013 guidelines (called in short “ART at CD4<500”): CD4≤500 cells/mm^3^ plus PMTCT option B+ (i.e., lifelong ART for all HIV-positive pregnant and breastfeeding women whatever their CD4 cell count); and, ii) the WHO 2015 guidelines (called in short “ART for all”): treatment for all whatever the CD4 cell count.
- For “ART at CD4<500” intervention, the scenario assumed that the ART coverage (among eligible individuals) increased from 41% to 55% in the 15–24 age group, from 56% to 75% in the 25–34 age group and from 77% to 85% in the 35–59 age group within the first four years (overall coverage: 81%). For “ART for all” intervention, the scenario assumed that the ART coverage (among eligible individuals) increased from 30% to 50% in the 15–24 age group, from 46% to 70% in the 25–34 age group and from 67% to 80% in the 35–59 age group within the first four years (overall coverage: 73%). To do this, a transition rate was added between compartments I_1_ and T and its value was supposed to increase linearly over the first year and finally be proportional to the value of the rate between compartments I_2_ and T. For “ART at CD4<500”, this transition rate was multiplied by the proportion of individuals with CD4 cell counts ≤500 cells/mm^3^ or pregnant or breastfeeding HIV-positive women (proportions stemming from the MHIPS data).
- Although HIV-positive individuals initiating ART were assumed to remain on ART during the whole period of simulation, we assumed that 93% of women and 92% of men on ART had a viral load <1000 copies/mL (and thus had reduced infectiousness). A sensitivity analysis was performed assuming an annual drop-out from ART of 1.5% [20] or 5% [21].
- **Voluntary medical male circumcision (VMMC).** Two scenarios were considered for this intervention. The first scenario (“non-targeted VMMC” scenario) assumed a slight increase in the proportion of circumcised HIV-negative men with age-specific targets: from 26% to 35% in the 15–24 age group, from 21% to 25% in the 25–34 age group, and from 19% to 20% in the 35–59 age group (overall proportion: from 22% to 30%). The second scenario (“targeted VMMC” scenario) focused on the 15–24 age group with a high increase in the proportion of circumcised HIV-negative men: from 26% to 70% (overall proportion: from 22% to 55%). In these scenarios, proportions were age-specific with a linear increase within the first four years and stable thereafter, and took ageing into account.
- **Pre-exposure prophylaxis (PrEP) in women.** Three scenarios were considered for this intervention: a) “non-targeted PrEP” scenario: use of PrEP at 15% in the 15–24 age group, 10% in the 25–34 age group, and 5% in the 35–59 age group (overall proportion: 10%); b) “targeted PrEP” scenario at low level: 20% of women aged 15–24 years use PrEP (overall proportion: 10%); and c) “targeted PrEP” scenario at medium level: 40% of women aged 15–24 years use PrEP (overall proportion: 20%). In these scenarios, proportions were age-specific with a linear increase within the first four years and stable thereafter, and took ageing into account.
- **Combined interventions.** “ART at CD4<500” and “ART for all” interventions were combined with VMMC and PrEP separately and jointly using both the non-targeted and age-targeted scenarios.
- These interventions were compared with respect to different outcomes: the incidence rate, the population viral load suppression, and the prevalence. Population viral load suppression was defined as the proportion of HIV-positive subjects with a viral load <1000 copies/mL (with the use of the compartment-specific proportions given in “Model and assumptions”). We reported reduction in incidence rate (in percentage) compared to the baseline rate (date at which the MHIPS was performed), unless otherwise specified. Moreover, ratios between the reduction in HIV incidence and the additional number of person-years on ART, or additional number of circumcisions or PrEP were calculated to compare results of “ART at CD4<500” and “ART for all”, and targeted and non-targeted scenarios.

## Results

With no additional interventions to the ones in place at the time of the survey (i.e. ART at CD4 < 350 and VMMC at 22% coverage among adult HIV-uninfected men), the HIV incidence rate would be reduced by 24% (from 2.25 to 1.70 per 100 person-years) by 4 years and by 25% (to 1.68 per 100 person-years) by 10 years. The reduction in HIV incidence would be higher in women than in men by 4 years (27% vs. 11% respectively compared to the baseline rate; Table 2). After a decrease, the HIV incidence in men would slighlty increase by 10 years due to a saturation effect given that ART was limited to HIV-positive women and men with CD4 < 350 cells/mm^3^. The reduction in HIV incidence would be higher in age groups 15–24 and 25–34 years than in the age group 35–59 years (31% and 29% vs. 18% respectively by 4 years, Table 2, and by 38% and 37% vs. 17% by 10 years).

**Table 2:** HIV incidence rates per 100 person-years (and percentage reduction compared to the baseline incidence rate), prevalence (%), and population viral load suppression (%) after four years of interventions

	Incidence rates						Overall prevalence	Overall population viral load suppression
	Overall (baseline: 2.25)	Men (baseline: 1.15)	Women (baseline: 3.28)	Aged 15–24 years (baseline: 1.76)	Aged 25–34 years (baseline: 4.58)	Aged 35–59 years (baseline: 1.56)		
No change^a^	1.70 (−24%)	1.01 (−11%)	2.40 (−27%)	1.21 (−31%)	3.24 (−29%)	1.28 (−18%)	26.5	65.1
ART at CD4<500 at:								
55%, 75%, 85%^b^								
+ Baseline VMMC	1.57 (−30%)	0.88 (−23%)	2.27 (−31%)	1.13 (−36%)	2.98 (−35%)	1.14 (−27%)	26.3	69.3
15–24 years: 26%								
25–34 years: 21%								
35–59 years: 19%								
+ Increased VMMC at:	1.50 (−34%)	0.77 (−32%)	2.23 (−32%)	1.08 (−39%)	2.81 (−39%)	1.13 (−27%)	26.3	69.4
15–24 years: 70%								
+ Increased VMMC at:	1.55 (−31%)	0.85 (−26%)	2.25 (−31%)	1.12 (−36%)	2.93 (−36%)	1.13 (−28%)	26.3	69.3
15–24 years: 35%								
25–34 years: 25%								
35–59 years: 20%								
ART for all at:								
50%, 70%, 80%^b^								
+ Baseline VMMC	1.44 (−36%)	0.77 (−33%)	2.12 (−35%)	1.05 (−40%)	2.74 (−40%)	1.02 (−34%)	26.2	72.8
15–24 years: 26%								
25–34 years: 21%								
35–59 years: 19%								
+ Increased VMMC at:	1.38 (−39%)	0.68 (−41%)	2.09 (−36%)	1.01 (−43%)	2.58 (−44%)	1.01 (−35%)	26.1	72.9
15–24 years: 70%								
+ Increased VMMC at	1.42 (−37%)	0.74 (−35%)	2.11 (−36%)	1.04 (−41%)	2.69 (−41%)	1.01 (−35%)	26.1	72.8
15–24 years: 35%								
25–34 years: 25%								
35–59 years: 20%								
ART for all at:								
50%, 70%, 80%^b^								
+ Increased PrEP at:	1.36 (−40%)	0.76 (−34%)	1.97 (−40%)	0.96 (−46%)	2.62 (−43%)	1.01 (−35%)	26.1	72.9
15–24 years: 20%								
+ Increased PrEP at:	1.28 (−43%)	0.75 (−35%)	1.82 (−45%)	0.86 (−51%)	2.50 (−45%)	1.00 (−36%)	25.9	73.1
15–24 years: 40%								
+ Increased PrEP at:	1.37 (−39%)	0.76 (−34%)	1.98 (−40%)	0.98 (−44%)	2.60 (−43%)	1.00 (−36%)	26.1	72.9
15–24 years: 15%								
25–34 years: 10%								
35–59 years: 5%								

^a^No change in baseline interventions: ART at CD4 < 350 and baseline VMMC (i.e. VMMC coverage at 26%, 21%, and 19% among age groups 15–24, 25–34, and 35–59 years respectively; see Methods section)

^b^For age groups 15–24 years, 25–34 years and 35–59 years respectively

Under this scenario, the prevalence would remain relatively stable over the simulation time (from 26.2% to 26.5% and 26.4% by 4 years and 10 years respectively) whereas the ART coverage among individuals with CD4 < 350 would increase from 77% to 91% and 93% by 4 and 10 years respectively, the ART coverage among all HIV-positive individuals from 53% to 63% and 65% by 4 years and 10 years respectively, and population viral load suppression (among all HIV-positive individuals) from 56% to 65% and 67% by 4 years and 10 years respectively.

Compared to the baseline rate, the targeted VMMC intervention would further reduce HIV incidence compared to the non-targeted VMMC intervention (reduction overall: 28% and 25% respectively by 4 years, 36% and 28% respectively by 10 years). Although the targeted VMMC scenario would provide greater reduction in HIV incidence than the non-targeted VMMC scenario (15% and 8% respectively compared “No change” scenario by 4 years), the latter would provide a greater reduction in incidence per additional number of circumcisions performed (32% per additional number of circumcisions performed).

Compared to the baseline rate, the targeted PrEP intervention at 40% among 15–24-year-old HIV-uninfected women would further reduce HIV incidence compared to the non-targeted PrEP intervention (reduction overall: 32% vs. 28% respectively by 4 years and 37% vs. 30% respectively by 10 years). The targeted scenario would provide a slightly greater reduction (3% by 4 years) in incidence per additional number of PrEP use. The targeted PrEP intervention at 20% among 15–24-year-old HIV-uninfected women had similar impacts to the non-targeted PrEP intervention.

Compared to the baseline rate, the “ART at CD4<500” intervention (i.e. initiation at CD4≤500 cells/mm^3^ plus PMTCT option B+, with baseline VMMC) would reduce HIV incidence by 30% overall by 4 years and 41% by 10 years. The reduction in HIV incidence would be higher in women than in men (31% vs. 23% by 4 years and 42% vs. 29% by 10 years). The reduction would be higher in the first two age groups (around 35% in the age groups 15–24 years and 25–34 years vs. 27% in the age group 35–59 years by 4 years and around 49% vs. 37% by 10 years). This intervention would lead to a population viral load suppression of 69% by 4 years and 75% by 10 years.

Combining “ART at CD4<500” intervention with VMMC targeted to young men would have a higher impact in reducing the baseline incidence compared to “ART at CD4<500” intervention with non-targeted VMMC overall (34% vs. 31% respectively) and among men (32% vs. 26%; Table 2). By 10 years, combination of “ART at CD4<500” intervention with VMMC targeted to young men would also have a higher impact in reducing the baseline incidence compared to “ART at CD4<500” intervention with non-targeted VMMC overall (49% vs. 43% respectively) and among men (45% vs. 33%).

The “ART for all” intervention would lead to a greater population viral load suppression (73% and 80% by 4 and 10 years respectively) and a greater impact on reducing HIV incidence rate (overall 36% and 51% by 4 and 10 years respectively). The reduction in HIV incidence would be slightly higher in women than in men (35% vs. 33% by 4 years and 51% vs. 44% by 10 years). The reduction would be higher in the first two age groups (around 40% in the age groups 15–24 and 25–34 years vs. 34% in the age group 35–59 years by 4 years and around 58% vs. 50% by 10 years). Compared to “No change” scenario, “ART at CD4<500” and “ART for all” would further reduce HIV incidence rate by 8% and 15% by 4 years, respectively. Dividing these reductions to the additional number of person-years on ART, “ART for all” would provide 2% greater reduction in incidence per additional number of person-years on ART compared to “ART at CD4<500”.

Combining “ART for all” intervention with VMMC targeted to young men would further reduce the incidence compared to “ART for all” intervention with non-targeted VMMC (overall reduction compared to baseline: 39% vs. 37% respectively by 4 years, and 57% vs. 53% respectively by 10 years). Combining “ART for all” intervention with PrEP targeted to young women would further reduce the incidence compared to “ART for all” intervention with non-targeted PrEP (overall: 40% and 43% depending on the targeted level vs. 39% by 4 years, and 55% and 59% depending on the targeted level vs. 54% by 10 years).

**Fig. 1:**
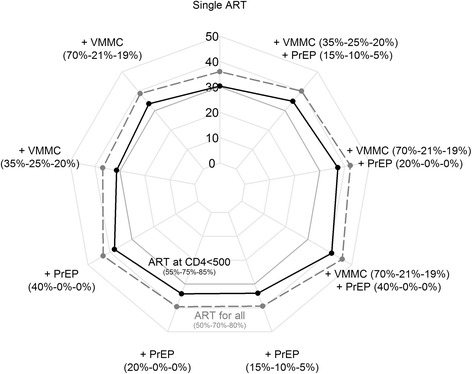
Reduction (%) in HIV incidence rate at 4 years compared to the baseline HIV incidence rate of “ART at CD4<500” or “ART for all” interventions and additional interventions. Percentages in brackets are the targeted values by age groups in men for VMMC intervention and in women for PrEP intervention

**Fig. 2:**
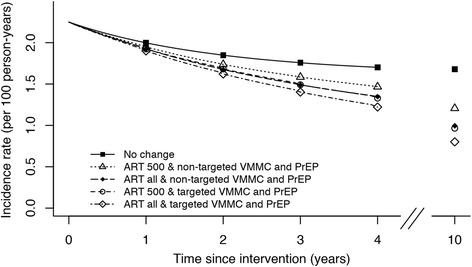
Overall HIV incidence rate for “No change” scenario and combined interventions. In this figure, the targeted PrEP scenario is the scenario with 40% of women aged 15–24 years using PrEP

Figure 1 shows the reduction in HIV incidence compared to the baseline rate for “ART at CD4<500” (black) or “ART for all” (grey) as a single intervention (top of the figure), combined to the targeted or non-targeted VMMC intervention (left), combined to the targeted or non-targeted PrEP intervention (bottom left), or combined to both VMMC and PrEP interventions with different scenarios. Combination of the three interventions (ART, VMMC, and PrEP) would have further impact on the overall incidence rate: from 35% to 41% reduction with “ART at CD4<500” and from 40% to 46% reduction with “ART for all” by 4 years depending on scenarios (Fig. 1 and Table 3). Combination of an ART intervention with age-targeted VMMC and/or PrEP interventions in young people would have the greatest impact on HIV incidence reduction (Fig. 2). Similar trends were observed by 10 years: combination of the three interventions (ART, VMMC, and PrEP) would have further impact on the overall incidence rate: from 46% to 57% reduction with “ART at CD4<500” and from 56% to 64% reduction with “ART for all” depending on scenarios.

**Table 3:** Reduction (%) in HIV incidence rate at 4 years compared to the baseline rate for an increased coverage of ART, VMMC and PrEP in different combinations

	No PrEP	+ PrEP 15–24 years: 40% 25–34 years: 0% 35–59 years: 0%	+ PrEP 15–24 years: 20% 25–34 years: 0% 35–59 years: 0%	+ PrEP 15–24 years: 15% 25–34 years: 10% 35–59 years: 5%
No change^a^	24%	32%	28%	28%
ART at CD4<500 at				
15–24 years: 55%				
25–34 years: 75%				
35–59 years: 85%				
+ Baseline VMMC	30%	38%	34%	34%
15–24 years: 26%				
25–34 years: 21%				
35–59 years: 19%				
+ Increased VMMC at	34%	41%	37%	37%
15–24 years: 70%				
+ Increased VMMC at	31%	39%	35%	35%
15–24 years: 35%				
25–34 years: 25%				
35–59 years: 20%				
ART for all at				
15–24 years: 50%				
25–34 years: 70%				
35–59 years: 80%				
+ Baseline VMMC	36%	43%	40%	39%
15–24 years: 26%				
25–34 years: 21%				
35–59 years: 19%				
+ Increased VMMC at	39%	46%	42%	42%
15–24 years: 70%				
+ Increased VMMC at	37%	44%	40%	40%
15–24 years: 35%				
25–34 years: 25%				
35–59 years: 20%				

^a^No change in baseline interventions: ART at CD4 < 350 and baseline VMMC (i.e. VMMC coverage at 26%, 21%, and 19% among age groups 15–24, 25–34, and 35–59 years respectively; see Methods section)

Reductions in HIV incidence were only slightly changed when varying the values of the mortality rates, and the comparisons between interventions remained unchanged (Additional file 1: Table S1).

The reduction in HIV incidence was lower when including a drop-out from ART in “No change” scenario (overall reduction compared to the baseline HIV incidence rate with an annual drop-out of 1.5% and 5%: 22% and 17% respectively by 4 years, 22% and 14% respectively by 10 years; Additional file 1: Table S2). The number of individuals to treat to achieve the target coverage for “ART at CD4<500” and “ART for all” would be higher when including a drop-out rate than without; while HIV incidence reduction was similar by 4 and 10 years.

## Discussion

We compared the potential impact of ART interventions and combined interventions on HIV incidence in a hyperendemic setting in KZN using age-specific targets for the ART, VMMC, and PrEP interventions, and age-targeted scenarios for the VMMC and PrEP interventions. We found that age-targeted interventions had a greater impact on incidence reduction than interventions without. Looking at the impact on the incidence of each intervention isolated, ART initiation regardless of CD4 count had the greatest impact. Our results are consistent with those from previous mathematical models applied to Sub-Saharan populations showing that substantial reduction in HIV incidence could be achieved with scale-up of ART (especially within a ‘test and treat’ strategy with extended ART eligibility) [20–28].

Recently, combination HIV prevention interventions have received increasing attention [29–31] although little is known about the dynamics of combining various interventions. Mathematical modelling has thus been using to assess the potential impact of combined interventions on HIV incidence in several settings [13, 32–38]. Our results showed that extended criteria for ART initiation in combination with moderate VMMC and PrEP coverage would further decrease incidence rate compared to ART as a single intervention. A recent modelling of data from KZN showed that HIV incidence rate would be reduced by around 60% within four years with a combination of four interventions: increasing the coverage of testing and counselling, reducing risky behaviour, increasing the coverage of VMMC, and increase ART coverage with extending eligibility to all HIV-positive individuals [37]. Cori et al. estimated that in South Africa a combination of increasing home-based voluntary testing and counselling, increasing VMMC coverage, and increasing ART coverage with a treat-all strategy over three years would lead to a 60% reduction [38]. Cremin et al. showed that combination of early ART, circumcision, and PrEP at relatively high levels would reduce HIV incidence in KZN as high as 84% over 10 years compared to the “status quo” (with ART eligibility at 200 CD4 cell count/mm^3^) [33].

Our results showed that, among the various scenarios considered, the combination of ART for all with PrEP targeted to 15–24-year-old women at 40% would be the most effective scenario to reduce overall incidence, incidence among women and among young adults. The most effective scenario to reduce incidence in men would be the combination of ART for all with VMMC at 70% among men aged 15–24 years. However, achieving these coverage rates may be difficult, the combination of ART for all with non-age-targeted VMMC or age-targeted PrEP at 20% coverage would be more feasible and also effective in reducing incidence rates.

These interventions would require extending ART eligibility to all HIV-positive individuals, requiring adequate prior coverage in each step of the care cascade. The latter is critical in a treatment-as-prevention interventions: the first results of the ANRS 12249 TasP trial in northern KZN did not show a statistically significant difference in HIV incidence over a four-year period between the intervention group (ART initiation irrespective of CD4) and the control group (ART initiation according to the national guidelines) which may be explained by the low linkage to care of newly diagnosed individuals in both arms (47% within 12 months) and the small difference in ART coverage between arms (45% in the intervention arm vs. 43% in the control arm) while ART eligibility should have been higher in the intervention arm than in the control arm [39]. In Mbongolwane and Eshowe, the achievements obtained by MSF and KZN department of Health interventions in population testing [40] and extension of ART eligibility from CD4 ≤350 to ≤500 cells/mm^3^ [40, 41] are promising for extending ART to all HIV-positive individuals. Indeed, program data showed acceptability from individuals with CD4 351–500 cells/mm^3^ to initiate and remain on ART (70% of individuals initiated on ART after testing positive with CD4 counts 351–500 and >80% retention in care at 12 months) and without negative effects for individuals with CD4 < 350 cells/mm^3^. However, strategies should be reinforced or implemented to ensure high adherence to ART to achieve high level of viral suppression; level of viral suppression among individuals on ART being a key parameter to ensure the success of a treatment-as-prevention strategy [34].

Our results showed that, over a four-year period, population viral load suppression would reach 69% under ART at CD4<500 and 73% under ART for all reaching UNAIDS targets (90%*90%*90%). These results have been obtained assuming steady proportions of viral load suppression over time among HIV-positive individuals, and particularly among those on ART. Few modelling studies have calculated this metric [13, 34], mainly due to lack of data on levels of viral suppression at population level. However, this is an interesting metric since it has been shown to be negatively associated with incidence [42–44] and can be estimated more easily than incidence in population. Moreover, this metric will be increasingly used with the current implementation of viral load monitoring in Sub-Saharan Africa.

The PrEP intervention was included here despite previous mixed results in Sub-Saharan Africa (discrepancies explained by differences in adherence levels) [45] and its costs. If PrEP is implemented, strategies should be developed to ensure high levels of adherence and therefore effectiveness in reducing HIV acquisition risk. New technologies currently assessed in clinical trials such as vaginal rings [46] or long-acting injectable ARV [47] could improve effectiveness levels.

Previous modelling studies showed that extending ART to higher CD4 count levels would be cost-effective [21, 48]. PrEP as an additional intervention, following extension of criteria for ART to higher CD4 count levels and increase in ART coverage (and increasing VMMC coverage), would be cost-effective when prioritized to key populations or persons with highest risks or incidence [28, 49–52]. High opportunity costs could be expected if PrEP is funded to the detriment of other more cost-effective HIV prevention interventions.

Our analysis had some limitations. First, our model assumed homogeneous (random) mixing among men and women between age groups. The reduction in HIV incidence could be overestimated if individuals in a given age group made relationships with individuals at higher risk whereas underestimated if individuals in a given age group made relationships with individuals at lower risk. Recent studies have reported complex age-mixing sexual patterns [53–55]. However, since the assumption of homogeneous mixing between age groups was made for each intervention, similar relative trends could be expected when comparing interventions. Second, our model does not account for non-heterosexual HIV transmission. However, in South Africa, overall HIV transmission does not seem to be substantially driven by men who have sex with men [56, 57], yet they can (directly or indirectly) benefit from the interventions modelled or focused interventions. Third, the steps of the cascade of care were implicitly modelled; however, the success of an ART intervention requires high levels in each step of the cascade of care and, as above-mentioned, strategies must be strengthened or implemented to reach high levels. Fourth, the development and transmission of drug resistance in HIV-positive individuals on ART or in those acquiring HIV while using PrEP were not modelled. Non-nucleoside reverse transcriptase inhibitor resistance levels were estimated at 14% among patients initiating and 38% among those re-initiating ART in South Africa [58]. These levels are likely to increase with the scale-up of ART and extension of ART eligibility criteria [59–61]. HIV drug resistance could substantially increase the number of AIDS deaths, the number of new infections, and ART program costs [62], but the benefits of ART interventions would not be counterbalanced by the increase in HIV drug resistance levels [63, 64]. Moreover, several modelling studies have showed that the majority of resistance cases would be rather due to ART than to PrEP, ART coverage being higher than PrEP coverage [65, 66]. South African ART guidelines recommended the use of viral load for monitoring patients on ART [67] which could allow early detection of failure and decrease resistance [68]. Observational data on the development and transmission of drug resistance in HIV-positive individuals on ART under extended ART eligibility would be of high interest. Lastly, we compared age-specific targets and age-targeted interventions. However, depending on the study area (e.g. a whole country or a district), interventions could target other population groups (e.g. by geography or risk group) and with the additional use of cost analyses [32, 35]. Moreover, we focused on the combination of ART, VMMC, and PrEP, although combination HIV prevention interventions include a wide range of interventions which could further reduce the number of new HIV infections.

## Conclusions

In Mbongolwane and Eshowe, implementation of the new South African guidelines for ART initiation (regardless of CD4 count) would accelerate reduction in incidence. Combined strategies, particularly if targeted to young people, would further reduce the HIV incidence in adult population.

## Additional file

Additional file 1: Technical appendix and supplementary results. (PDF 639 kb)

## Abbreviations

AIDS: Acquired immunodeficiency syndrome
ART: Antiretroviral therapy
HIV: Human immunodeficiency virus
KZN: KwaZulu-Natal
MHIPS: Mbongolwane and Eshowe HIV Impact in Population Survey
MSF: Médecins Sans Frontières (Doctors Without Borders)
PMTCT: Prevention of mother-to-child transmission
PrEP: Pre-exposure prophylaxis
UNAIDS: Joint United Nations Programme on HIV and AIDS
VMMC: Voluntary medical male circumcision
WHO: World Health Organization
