# Supplementary material for: Combined interventions to reduce HIV incidence in KwaZulu-Natal: a modelling study
Source: BMC Infect Dis. 2017 Jul 26;17:522. doi: 10.1186/s12879-017-2612-5 (PMC5530541; doi:10.1186/s12879-017-2612-5)
Supplement: Additional file 1: — Technical appendix and supplementary results. (PDF 639 kb) [file 12879_2017_2612_MOESM1_ESM.pdf]

Appendix

Combined interventions to reduce HIV incidence in KwaZulu-Natal: a modelling study

Stéphanie Blaizot, Helena Huerga, Benjamin Riche, Tom Ellman, Amir Shroufi, Jean-François Etard, René Ecochard

Mathematical model

A detailed description of the mathematical model and the estimation method of the model parameters can be found in Reference [1]. Here, we described briefly the main characteristics of the mathematical model.

The model, shown in Figure S1, describes HIV transmission, the untreated-disease progression, and ART use in a heterosexual population. It splits the population into compartments according to sex, age, and HIV status. The population of infected individuals was split into three compartments according to the CD4 cell count and the ART status: 1) Compartment I_1: untreated HIV-positive individuals with CD4 cell count >350 cells/mm^3; 2) Compartment I_2: untreated HIV-positive individuals with CD4 cell count ≤350 cells/mm^3 (immunosuppressed individuals); and, 3) Compartment T: HIV-positive individuals under ART. An additional Compartment S was dedicated to HIV-negative (or susceptible) individuals.

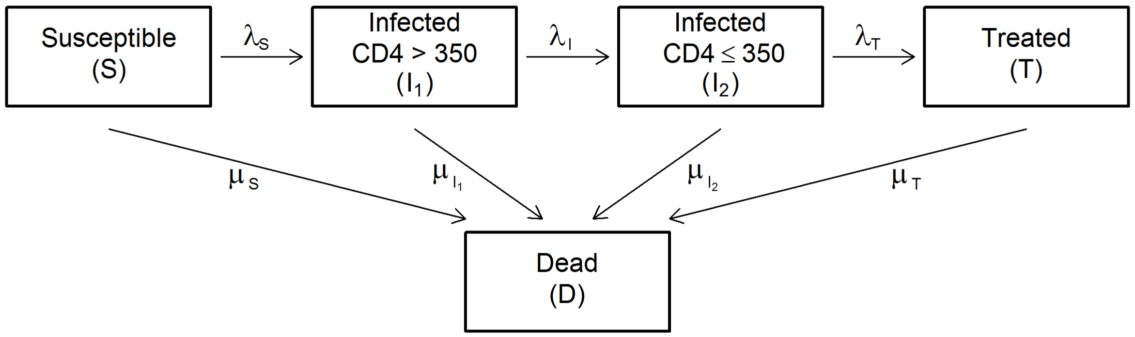

Figure S1. Simplified diagram of the model

Notation

S: susceptible individuals of the studied population

I_1: HIV-positive and untreated individuals with CD4 cell count >350 cells/mm3

I_2: HIV-positive and untreated individuals with CD4 cell count ≤350 cells/mm3

T: HIV-positive individuals on ART

D: deceased individuals

λ_S: force of infection

λ_I: immunosuppression rate

λ_T: treatment rate

μ_S: mortality rate of individuals in compartment S

μ_{I1}: mortality rate of individuals in compartment I_1

μ_{I2}: mortality rate of individuals in compartment I_2

μ_T: mortality rate of individuals in compartment T

Differential equations

We formulated the predictive model as a system of sex and age-specific (a = 15, ..., 59 years) differential equations:

$$\begin{cases} \dfrac{dS_{Sex,a}}{dt} = -\lambda_{S,Sex,a}\, S_{Sex,a} - \mu_{S,Sex,a}\, S_{Sex,a} - \nu\, S_{Sex,a} + \nu\, S_{Sex,a-1} \\ \dfrac{dI_{1Sex,a}}{dt} = \lambda_{S,Sex,a}\, S_{Sex,a} - \left(\mu_{I_1,Sex,a} + \lambda_{I,Sex,a}\right) I_{1Sex,a} - \nu\, I_{1Sex,a} + \nu\, I_{1Sex,a-1} \\ \dfrac{dI_{2Sex,a}}{dt} = \lambda_{I,Sex,a}\, I_{1Sex,a} - \left(\mu_{I_2,Sex,a} + \lambda_{T,Sex,a}\right) I_{2Sex,a} - \nu\, I_{2Sex,a} + \nu\, I_{2Sex,a-1} \\ \dfrac{dT_{Sex,a}}{dt} = \lambda_{T,Sex,a}\, I_{2Sex,a} - \mu_{T,Sex,a}\, T_{Sex,a} - \nu\, T_{Sex,a} + \nu\, T_{Sex,a-1} \end{cases}$$

Ageing was considered through the last two terms of each equation; v being the rate at which an individual moves from one age class to another. For the first age class (15 years), ageing was taken into account as follows: at one year intervals, a fixed number of individuals were put into compartment S.

The force of infection is frequency-dependent; it included HIV prevalence in the opposite sex weighted by the infectiousness of HIV-positive individuals. This infectiousness (probability of transmitting the virus) depends on the viral load. This force of infection may then be written as follows:

$$\lambda_{S,Sex,a} = \tilde{\beta}_{Sex,a} \left(\frac{J_{OppositeSex}}{N_{OppositeSex}} \right) \zeta_{Sex,a}$$

with:

$\tilde{\beta}_{Sex,a}$ the transmission parameter;

$\zeta_{Sex,a} = \left(1 - \varphi_{Sex,a}\right) + \gamma\varphi_{Sex,a}$ allows us to take into account that some individuals, in proportion φ, have a different susceptibility (reduced by γ) due to, for example, circumcision (in men);

$$J_{OppositeSex} = I_{1OppositeSex}\left[\left(1 - propVLlow_{I_1,OppositeSex}\right) + propVLlow_{I_1,OppositeSex} \cdot \varepsilon\right] + I_{2OppositeSex}\left[\left(1 - propVLlow_{I_2,OppositeSex}\right) + propVLlow_{I_2,OppositeSex} \cdot \varepsilon\right] + T_{OppositeSex}\left[\left(1 - propVLlow_{T,OppositeSex}\right) + propVLlow_{T,OppositeSex} \cdot \varepsilon\right]$$

$propVLlow_{X,OppositeSex}$ is the (baseline) proportion of individuals with a viral load below 1000 copies/mL in the compartment X and ε is the reduction of infectiousness in individuals with a viral load below 1000 copies/mL.

Parameters

The model parameters are the following: i) the force of infection (λ_S); ii) the "immunosuppression rate" (λ_I); i.e., the rate at which an individual moves from > 350 to ≤ 350 cells/mm^3 CD4 cell count; iii) the "treatment rate" (λ_T) or the ART initiation rate; and, iv) the mortality rate (μ).

Estimating the model parameters included three stages. First, we used the HIV status, the ART status, and the CD4 cell count at the time of the survey to assign each individual to one of the above-cited compartments. Second, we derived individuals' states (as described by the compartments) during the past year from the individuals' histories (self-reported dates of first positive HIV test, last HIV test and its result, and ART initiation) and CD4 measurement. We used only the previous year to minimize the recall bias and avoid making too strong assumptions about individuals' histories. Third, we calculated the number of transitions between pairs of compartments and the time spent by each individual in each compartment.

The initial compartment sizes (i.e., the number of individuals in each compartment) stemmed from the MHIPS survey and applied on the 2011 census (Figures S2-S3).

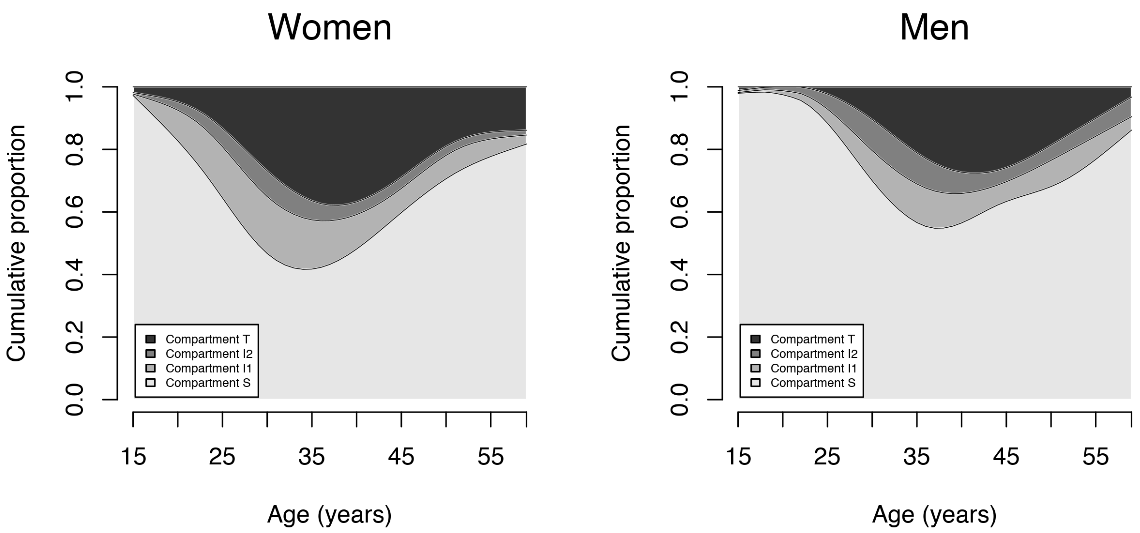

Figure S2. Initial cumulative proportion of individuals in each compartment by sex and age

S: susceptible individuals; I1: HIV-positive and untreated individuals with CD4 cell count >350 cells/mm^3; I2: HIV-positive and untreated individuals with CD4 cell count ≤350 cells/mm^3; T: HIV-positive individuals on ART.

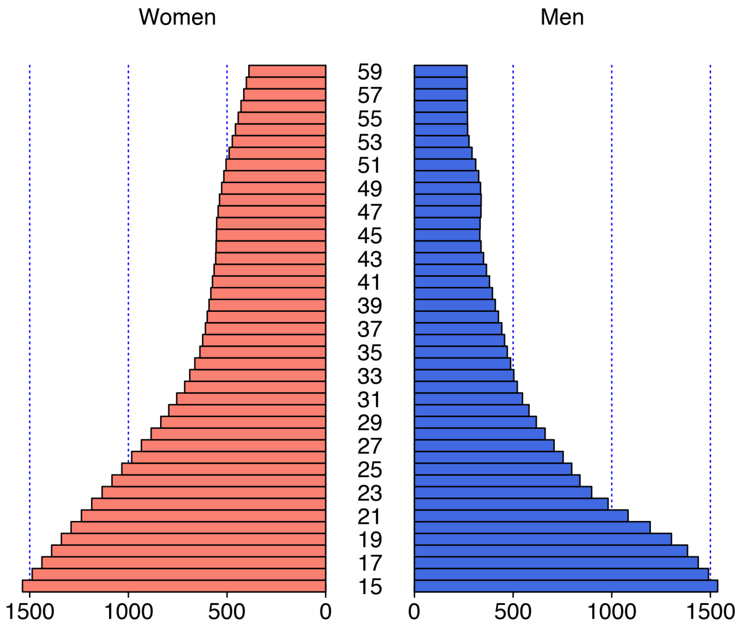

Figure S3. Initial population size by sex and age (15-59 years)

Sensitivity analyses

We assumed that individuals in compartments S, I_1, and T had the same risk of death whereas individuals in compartment I_2 had an additional risk of death due to AIDS, and used mortality rates from another region in KwaZulu-Natal [2]. A sensitivity analysis was performed to assess the impact of this assumption on results: the mortality rates in individuals in compartments I_1 and T were varied with values being one tenth, one fifth, or half those in individuals in compartment I_2.

Table S1. Percentage reduction of incidence rate compared to the baseline incidence rate depending on values of mortality rates after four years of interventions

	Overall	Men	Women	Aged 15-24 years	Aged 25-34 years	Aged 35-59 years
**No change** [a]	24-29%	12-17%	27-32%	31-36%	29-34%	18-23%
**ART at CD4<500 at** 55%, 75%, 85% + Baseline VMMC (26%,21%,19%)	30-35%	24-28%	31-35%	35-40%	35-39%	27-31%
+ Increased VMMC at 70%, 21%, 19%	33-38%	33-36%	32-36%	38-43%	39-43%	27-32%
+ Increased VMMC at 35%, 25%, 20%	31-36%	26-30%	31-36%	36-40%	36-40%	28-32%
**ART for all at** 50%, 70%, 80% + Baseline VMMC (26%,21%,19%)	36-40%	33-37%	35-39%	40-44%	40-44%	34-38%
+ Increased VMMC at 70%,21%,19%	39-43%	41-44%	36-40%	43-46%	43-47%	35-39%
+ Increased VMMC at 35%, 25%,20%	37-41%	36-39%	35-40%	41-45%	41-45%	35-39%
**ART for all at** 50%, 70%, 80%						
+ Increased PrEP at 20%,0%,0%	39-43%	34-38%	40-44%	45-49%	43-46%	35-39%
+ Increased PrEP at 40%, 0%, 0%	43-47%	35-38%	44-48%	51-54%	45-49%	36-39%
+ Increased PrEP at 15%, 10%, 5%	39-43%	34-38%	39-43%	44-48%	43-47%	36-40%

[a] No change in baseline interventions: ART at CD4<350 and baseline VMMC (i.e. VMMC coverage at 26%, 21%, and 19% among age groups 15-24, 25-34, and 35-59 years respectively; see Methods section)

The results of the sensitivity analysis assuming an annual drop-out from ART of 1.5% [3] or 5% [4] are shown in Table S2.

Table S2. Percentage reduction of incidence rate compared to the baseline incidence rate after four years of interventions depending on the value of annual drop-out from ART (left %: drop-out of 5%, right %: drop-out of 1.5%/year).

	Overall	Men	Women	Aged 15-24 years	Aged 25-34 years	Aged 35-59 years
No change [a]	17-22%	3-9%	19-24%	24-29%	22-27%	10-15%
**ART at CD4<500** at 55%, 75%, 85%						
+ Baseline VMMC (26%,21%,19%)	27-29%	23-23%	27-29%	32-34%	32-33%	25-26%
+ Increased VMMC at 70%, 21%, 19%	31-32%	32-32%	28-30%	35-37%	36-37%	26-26%
+ Increased VMMC at 35%, 25%, 20%	28-30%	26-25%	27-30%	33-35%	33-35%	26-26%
**ART for all** at 50%, 70%, 80%						
+ Baseline VMMC (26%,21%,19%)	34-35%	33-33%	33-34%	38-39%	38-40%	33-34%
+ Increased VMMC at 70%,21%,19%	37-38%	40-41%	34-35%	41-42%	42-43%	34-34%
+ Increased VMMC at 35%, 25%,20%	35-36%	35-35%	33-35%	39-40%	39-41%	34-35%
**ART for all** at 50%, 70%, 80%						
+ Increased PrEP at 20%,0%,0%	38-39%	33-34%	38-39%	44-45%	41-42%	34-35%
+ Increased PrEP at 40%, 0%, 0%	41-42%	34-35%	42-44%	49-50%	44-45%	34-35%
+ Increased PrEP at 15%, 10%, 5%	37-39%	33-34%	37-39%	42-44%	41-43%	34-35%

[a] No change in baseline interventions: ART at CD4<350 and baseline VMMC (i.e. VMMC coverage at 26%, 21%, and 19% among age groups 15-24, 25-34, and 35-59 years respectively; see Methods section)

References

1. Blaizot S, Riche B, Maman D, Mukui I, Kirubi B, Etard JF, Ecochard R: **Estimation and Short-Term Prediction of the Course of the HIV Epidemic Using Demographic and Health Survey Methodology-Like Data**. *PLoS One* 2015, **10**(6):e0130387.
2. Nyirenda M, Hosegood V, Barnighausen T, Newell ML: **Mortality levels and trends by HIV serostatus in rural South Africa**. *AIDS* 2007, **21 Suppl 6**:S73-79.
3. Granich RM, Gilks CF, Dye C, De Cock KM, Williams BG: **Universal voluntary HIV testing with immediate antiretroviral therapy as a strategy for elimination of HIV transmission: a mathematical model**. *Lancet* 2009, **373**(9657):48-57.
4. Eaton JW, Menzies NA, Stover J, Cambiano V, Chindelevitch L, Cori A, Hontelez JAC, Humair S, Kerr CC, Klein DJ *et al*: **Health benefits, costs, and cost-effectiveness of**

earlier eligibility for adult antiretroviral therapy and expanded treatment coverage: a combined analysis of 12 mathematical models. *Lancet Glob Health* 2014, **2**(1):E23-E34.
